# Two morbilliviruses implicated in bottlenose dolphin epizootics.

**DOI:** 10.3201/eid0203.960308

**Published:** 1996

**Authors:** J K Taubenberger, M Tsai, A E Krafft, J H Lichy, A H Reid, F Y Schulman, T P Lipscomb

**Affiliations:** Division of Molecular Pathology, Department of Cellular Pathology, Armed Forces Institute of Pathology, Washington, D.C. 20306-6000, USA.


### Two Morbilliviruses Implicated in Bottlenose Dolphin Epizootics

Sequence analysis was performed on viral RNA isolated from bottlenose dolphins (*Tursiops truncatus*) that died during two chronologically and geographically separate epizootics in North America. Both dolphin morbillivirus (DMV) and porpoise morbil-livirus (PMV) were detected in bottlenose dolphins that died during the 1987 U.S. Atlantic coast epizootic. Our results indicate not only that these viruses are not species specific, but also that both viruses were present in North America before outbreaks in the Mediterranean and Irish Seas. Samples taken along the Atlantic coast showed a statistically significant trend with DMV in the north and an increasing incidence of PMV in samples isolated farther south. In the 1993 Gulf of Mexico epizootic, only PMV was detected in bottlenose dolphins that died. Thus, DMV and PMV are implicated as the causes of the earliest known aquatic mammal morbilliviral outbreak, the U.S. Atlantic coast epidemic; PMV is implicated in the Gulf of Mexico epidemic. The presence of two pathogenic morbilliviruses that may circulate together or separately complicates the epidemiology of cetacean morbilliviral diseases.

The only morbilliviruses known before 1989 were human measles virus, canine distemper virus, rinderpest, and peste-des-petits-ruminants virus (1). Recently, newly characterized morbilliviruses have been shown to be epizootic-associated pathogens in pinnipeds and cetaceans. Phocine distemper virus (2) was associated with a massive epizootic of harbor seals (*Phoca vitulina*) in northwestern Europe (3) in 1988. During the harbor seal phocine distemper virus outbreak, PMV was isolated from harbor porpoises (*Phocoena phocoena*) that died along the Irish coast (4). DMV (5), was isolated during an epizootic of striped dolphins (*Stenella coeruleoalba*) in the Mediterranean Sea (6,7) in 1990-92.

Between June of 1987 and May of 1988, a morbillivirus epizootic caused a tenfold increase in bottlenose dolphin stranding along the U.S. Atlantic coast from New Jersey to Florida (8,9). More than half of the in-shore population of bottlenose dolphins in this area may have died. Morbillivirus-related strandings of bottlenose dolphins along the Gulf of Mexico coasts of Alabama, Mississippi, and Texas were also observed from October 1993 through April 1994 (10,11).

Using reverse transcriptase–polymerase chain reaction (RT-PCR), we examined tissue lysates made from formalin-fixed paraffin-embedded lung tissue from stranded dolphins from the Atlantic epizootic and frozen unfixed lung tissue from the Gulf of Mexico epizootic (11,12). RT-PCR-positive cases were subsequently characterized by sequence analysis of segments of the morbillivirus P gene in regions with the greatest diversity between DMV and PMV.

We sequenced segments of the morbillivirus P gene from 29 dolphin specimens (1 striped and 28 bottlenose dolphins) from the 1987-88 Atlantic epizootic; sequence sufficient for viral identification was obtained in 25 (86%) of 29 cases. Sequence was also obtained from 7 (37%) of 19 bottlenose dolphin specimens stranded in Texas during the Gulf of Mexico epizootic. Because of the advanced state of postmortem decomposition of most of these specimens, histologic and immunophenotypic analysis was not possible (13). Data (stranding date, location, species, and morbillivirus sequence analysis results) for these 32 cases are shown in Table 1. In addition, P gene sequence was obtained from formalin-fixed paraffin-embedded lung tissue from four of four striped dolphins from the 1990 Mediterranean Sea epizootic and from one harbor porpoise recovered off the coast of Northern Ireland in 1988 during the harbor seal epizootic (Table 1).

Morbillivirus P gene sequencing allowed viral identification of 37 stranded cetaceans from four geographically and chronologically separate epizootics. In the U.S. Atlantic Coast 1987-88 epizootic, mixed infection was present involving both recognized cetacean morbilliviruses, DMV and PMV (Table 1). Twelve animals (including the striped dolphin) were infected with DMV, nine with PMV, and four with both. From the later Gulf of Mexico morbillivirus-associated epizootic, only PMV was identified. The morbillivirus infecting striped dolphins in the Mediterranean Sea epizootic was identified as DMV (6). All four striped dolphin specimens examined in this study were typed as DMV-infected. A harbor porpoise stranded on the coast of Northern Ireland during the harbor seal epizootic in northwestern Europe was typed as PMV-infected, consistent with the published analysis of these animals (14).

Partial P gene nucleotide sequence data are presented with published DMV and PMV P gene sequences (Figure 1). Sequence data from the PMV-infected cases (Atlantic epizootic cases, Gulf of Mexico epizootic cases, Irish harbor porpoise)

Table 1. Case summary

| Date found | Stranding by state | Dolphin species | Morbillivirus sequence[a] |
|---|---|---|---|
| 4 Aug. 1987 | New Jersey | *Tursiops truncatus* | DMV |
| 5 Aug. 1987 | New Jersey | *T. truncatus* | DMV |
| 9 Aug. 1987 | New Jersey | *T. truncatus* | DMV |
| 11 Aug. 1987 | New Jersey | *T. truncatus* | DMV |
| 23 Aug. 1987 | New Jersey | *T. truncatus* | DMV + PMV |
| 3 Sep. 1987 | New Jersey | *Stenella coeruloalba* | DMV |
| 5 Sep. 1987 | New Jersey | *T. truncatus* | DMV + PMV |
| 6 Sep. 1987 | New Jersey | *T. truncatus* | PMV |
| 14 Aug. 1987 | Virginia | *T. truncatus* | PMV |
| 14 Aug. 1987 | Virginia | *T. truncatus* | DMV + PMV |
| 15 Aug. 1987 | Virginia | *T. truncatus* | PMV |
| 26 Aug. 1987 | Virginia | *T. truncatus* | DMV + PMV |
| 29 Aug. 1987 | Virginia | *T. truncatus* | PMV |
| 29 Aug. 1987 | Virginia | *T. truncatus* | DMV |
| 4 Sep. 1987 | Virginia | *T. truncatus* | DMV |
| 5 Oct. 1987 | Virginia | *T. truncatus* | DMV |
| 6 Oct. 1987 | Virginia | *T. truncatus* | DMV |
| 7 Oct. 1987 | Virginia | *T. truncatus* | DMV |
| 21 Dec. 1987 | Florida (Atlantic Coast) | *T. truncatus* | PMV |
| 21 Dec. 1987 | Florida (Atlantic Coast) | *T. truncatus* | PMV |
| 1 Jan. 1988 | Florida (Atlantic Coast) | *T. truncatus* | PMV |
| 18 Jan. 1988 | Florida (Atlantic Coast) | *T. truncatus* | PMV |
| 9 Feb. 1988 | Florida (Atlantic Coast) | *T. truncatus* | DMV |
| 9 Feb. 1988 | Florida (Atlantic Coast) | *T. truncatus* | PMV |
| 10 Feb. 1988 | Florida (Atlantic Coast) | *T. truncatus* | DMV |
| 4 Apr. 1993 | Texas | *T. truncatus* | PMV |
| 4 Apr. 1993 | Texas | *T. truncatus* | PMV |
| 4 Apr. 1993 | Texas | *T. truncatus* | PMV |
| 11 Apr. 1993 | Texas | *T. truncatus* | PMV |
| 15 Apr. 1993 | Texas | *T. truncatus* | PMV |
| 19 Apr. 1993 | Texas | *T. truncatus* | PMV |
| 21 Apr. 1993 | Texas | *T. truncatus* | PMV |
| 1991 | Mediterranean Sea | *S. coeruloalba* | DMV |
| 1991 | Mediterranean Sea | *S. coeruloalba* | DMV |
| 1991 | Mediterranean Sea | *S. coeruloalba* | DMV |
| 1991 | Mediterranean Sea | *S. coeruloalba* | DMV |
| 1988 | Northern Ireland | *Phocoena phocoena* | PMV |

[a] RNA isolation, RT-PCR, and cycle sequencing were performed (11,20).

showed no within-group variation. Data from all cases showed two single nucleotide changes from the published PMV sequence (12), at positions 206 and 209 (aligned with the measles virus P gene sense mRNA). Likewise, no sequence variation was noted for the DMV-infected animals (Atlantic and Mediterranean epizootics). However, a two-nucleotide inversion, at coding positions 206-207, was consistently noted when compared to the published DMV sequence (12) (Figure). In four cases from the Atlantic epizootic cycle sequencing indicated simultaneous infection with DMV and PMV. To confirm that the two different sequences were present simultaneously, PCR product was cloned, and plasmid DNA from individual colonies was sequenced. In each of the four doubly infected cases, approximately half the clones contained the DMV sequence, and half contained the PMV sequence. No clones containing hybrid sequences were found. Since PMV and DMV cause non-species-specific infections, more appropriate designations for these viruses might be cetacean mor-billivirus 1 for PMV (the first detected cetacean morbillivirus [4]) and cetacean morbillivirus 2 for DMV.

Bottlenose dolphins of the U.S. Atlantic coast migrate seasonally (15). The 1987-88 epizootic moved south from New Jersey to the east coast of Florida with the southerly fall migration. In-shore bottlenose dolphins rarely travel around the southern tip of the Florida peninsula (Randall Wells, pers. comm.), which explains the end of the 1987 epizootic on the east coast of Florida in May of 1988. Sequences were obtained from dolphins stranded during this epizootic in three U.S. states, New Jersey, Virginia, and Florida. From the Gulf of Mexico epizootic, which occurred 5 years later, sequence data from seven dolphins stranded in Texas were obtained. A significant trend (chi square for trends [16], $p$ = 0.00023) was noted among the specimens analyzed from the New Jersey, Virginia, and Florida Atlantic coasts in which the percentage of animals infected with DMV decreased, while PMV infection increased (Table 2). In the 1993 die-off, 100% of the animals from which sequence information was obtained were PMV-infected. The epidemiologic significance of this finding is unknown. It is possible that dolphins of the southern Atlantic coast and those in the Gulf of Mexico were more susceptible to infection with (or had an increased death rate from) PMV than with DMV.

To test whether enzootic morbillivirus infection could be detected in bottlenose dolphins, RT-PCR was performed on formalin-fixed paraffin-embedded tissue samples from 11 dolphins stranded along the U.S. Atlantic coast from 1974 to 1985 (before the 1987 epizootic). None of the dolphins had histologic or immunohistochemical evidence of morbillivirus infection (9). Amplifiable RNA was obtained from samples from eight animals, and all were negative for morbillivirus. Tissue specimens from seven bottlenose dolphins stranded on the Florida coast of the Gulf of Mexico in the interval between the Atlantic and Gulf of Mexico epizootics were also ex-

```
                          176                                                 215
Published DMV             ATCTGCTCCC   AGGATTAAGG   TCGAGAGATC   TGCTGACGTT
Atlantic/Mediter. DMV     ----------   ----------   ----------   GT--------
Atlantic/Gulf/Irish PMV   ----------   ----------   ----------   AT-C--T---
Published PMV             ----------   ----------   ----------   -T----T---

                          216                                                 255
Published DMV             GAGACTATAA   GCAGTGAAGA   GCTACAAGGA   CTGATTAGAT
Atlantic/Mediter. DMV     ----------   ----------   ----------   ----------
Atlantic/Gulf/Irish PMV   ----------   -------G--   A--T------   -----C----
Published PMV             ----------   -------G--   A--T------   -----C----

                          256                                                 295
Published DMV             CTCAGAGTCA   AAAACATAAT   GGATTTGGAG   TAGACAGATT
Atlantic/Mediter. DMV     ----------   ----------   ----------   ----------
Atlantic/Gulf/Irish PMV   ------AC--   C--G--G---   -----C----   G-----A--C
Published PMV             ------AC--   C--G--G---   -----C----   G-----A--C

                          296          313
Published DMV             CCTAAAGGTC   CCACCAAT
Atlantic/Mediter. DMV     ----------   --------
Atlantic/Gulf/Irish PMV   TT--------   --------
Published PMV             TT--------   --------
```

Figure. Partial nucleotide sequence of DMV and PMV morbillivirus P gene compared with published DMV and PMV sequences (12). Primer sequences used were 5'-ATC TGC TCC CAG GAT TAA GGT CGA-3' (forward) and 5'-CGG GAT TGG TGG GAC CTT TA-3' (reverse). RT-PCR was performed (11). PCR products were cycle sequenced (20) or cloned into PDK101 (21) and sequenced by using T7 Sequenase (Amersham Corporation, Arlington Heights, IL) according to the manufacturer's instructions.

amined by RT-PCR for morbillivirus. None of these animals had histologic or immunohistochemical evidence of morbillivirus infection (data not shown). However, one of these samples was weakly positive for the morbillivirus P gene 78 bp product (11). Sequence information, however, could not be obtained from this sample. This result suggests the possibility of an enzootic infection in dolphins along the U.S. coast after the initial 1987 morbillivirus epizootic.

Serum antibodies to canine distemper virus were detected in specimens of 6 of 13 free-ranging bottlenose dolphins during the U.S. Atlantic epizootic in 1987 (17), indicating exposure to morbillivirus. A recent study by Duignan, et al. (18) presented serologic evidence of morbillivirus infection in 11 of 15 cetacean species in the western Atlantic since 1986.

Table 2. Percentage of DM-V and PMV-infected animals by location

| Stranding location | No. dolphins | No. DMV infected (%) | No. PMV infected (%) |
|---|---|---|---|
| Atlantic, New Jersey | 8 | 7 (70)[a] | 3 (30)[a] |
| Atlantic, Virginia | 10 | 7 (58)[a] | 5 (42)[a] |
| Atlantic, Florida | 7 | 2 (29) | 5 (71) |
| Gulf of Mexico, Texas | 7 | 0 | 7 (100) |

[a] Including the four cases of simultaneous DMV and PMV infection.

Virus neutralizing titers were higher against DMV and PMV than against peste-des-petits ruminants virus, phocine distemper virus, or canine distemper virus. Enzootic morbillivirus infection of two species of pilot whale (*Globicephala melas* and *G. macrorhynchus*) was recently demonstrated in animals in the western Atlantic (19), with the earliest titer from a pilot whale stranded in 1982. Neutralizing antibody titers in these species were also higher against DMV and PMV, and were observed in 108 of 125 animals tested. However, confirmation of active infection by viral sequence analysis was not performed in either of these studies. It is, therefore, possible that the pilot whale mass strandings reported in 1982 and 1986 were morbillivirus induced, but confirmation of this hypothesis would require histologic and/or viral sequence confirmation.

Geographically and temporally distinct morbillivirus die-offs, as well as the evidence presented in this study that the two previously described cetacean morbilliviruses are not species-specific, raise many questions about the epidemiology of this family of viruses. The viruses implicated in the 1988 European porpoise deaths and 1990 Mediterranean dolphin epizootic were present in bottlenose dolphins of the 1987 U.S. die-off. Duignan, et al. hypothesized that pilot whales enzootically infected with morbilliviruses could act as long-distance vectors between America and Europe (19). Confirmation would require sequence analysis of pilot whale samples collected in both regions. The distribution of DMV and PMV infection in the U.S. epizootics suggests that the later epizootic may have been initiated by rare contact between PMV-infected Atlantic dolphins and immunologically naive Gulf dolphins. Further investigation of serum antibody titers, PCR evidence of enzootic infection, and sequence identification of morbillivirus species are required for the better understanding of these epizootics.

### Acknowledgments

This study was partially supported by a grant from the Center for Marine and Estuarine Disease Research, U.S. Environmental Protection Agency. We thank S. Kennedy, M. Domingo, the U.S. National Marine Fisheries Service, and the U.S. Marine Mammal Stranding Networks for providing samples.

**Jeffery K. Taubenberger, M.D., Ph.D.,* Mark Tsai, M.S.,* Amy E. Krafft, Ph.D.,* Jack H. Lichy, M.D., Ph.D.,* Ann H. Reid, M.A.,* F. Yvonne Schulman, D.V.M.,† and Thomas P. Lipscomb, D.V.M.†**

*Division of Molecular Pathology, Department of Cellular Pathology, and †Department of Veterinary Pathology, Armed Forces Institute of Pathology, Washington, DC 20306-6000 USA

### References

1. Diallo A. Morbillivirus group: genome organization and proteins. Vet Microbiol 1990;23:155-63.
2. Haas L, Subbarao SM, Harder T, Liess B, Barrett T. Detection of phocid distemper virus RNA in seal tissues using slot hybridization and the polymerase chain reaction amplification assay: genetic evidence that the virus is distinct from canine distemper virus. J Gen Virol 1991; 72:825-32.
3. Osterhaus ADME, Groen J, Spijkers HEM, Broeders HWJ, UytdeHaag FGCM, de Vries P, et al. Mass mortality in seals caused by a newly discovered virus-like morbillivirus. Vet Microbiol 1990;23:343-50.
4. McCullough SJ, McNeilly F, Allan GM, Kennedy S, Smyth JA, Cosby SL, et al. Isolation and characterization of a porpoise morbillivirus. Arch Virol 1991;118:247-52.
5. Osterhaus ADME, Visser IKG, de Swart RL, van Bressem M-F, van de Bildt MWG, Örvell C, et al. Morbillivirus threat to Mediterranean monk seals? Vet Rec 1992;130:141-2.
6. Domingo M, Ferrer L, Pumarola M, Marco A, Plana J, Kennedy S, et al. Morbillivirus in dolphins. Nature 1990;348:21.
7. Van Bressem M-F, Visser IKG, de Swart RL, Örvell C, Stanzani L, Androukaki E, et al. Dolphin morbillivirus infection in different parts of the Mediterranean Sea. Arch Virol 1993;129:235-42.
8. Lipscomb TP, Schulman FY, Moffett D, Kennedy S. Morbilliviral disease in Atlantic bottlenose dolphins (*Tursiops truncatus*) from the 1987-88 epizootic. J Wildl Dis 1994;30:567-71.
9. Schulman FY, Lipscomb TP, Moffett D, Krafft AE, Lichy JH, Tsai MM, et al. Reevaluation of the 1987-88 Atlantic coast bottlenose dolphin (*Tursiops truncatus*) mortality event with histolgic, immunohistochemical, and molecular evidence for a morbilliviral etiology. In preparation, 1996.
10. Lipscomb TP, Kennedy S, Moffett D, Ford BK. Morbilliviral disease in an Atlantic bottlenose dolphin (*Tursiops truncatus*) from the Gulf of Mexico. J Wildl Dis 1994;30:572-6.
11. Krafft AE, Lichy JH, Lipscomb TP, Klaunberg BA, Kennedy S, Taubenberger JK. Postmortem diagnosis of morbillivirus infection in bottlenose dolphins (*Tursiops truncatus*) in the Atlantic and Gulf of Mexico Epizootics by a polymerase chain reaction-based assay. J Wildl Dis 1995;31:410-5.
12. Barrett T, Visser IKG, Mamaev L, Goatley L, van Bressem M-F, Osterhaus ADME. Dolphin and porpoise morbil-liviruses are genetically distinct from phocine distemper virus. Virology 1993;193:1010-2.
13. Lipscomb TP, Kennedy S, Moffett D, Krafft AE, Klaunberg BA, Lichy JH, et al. Morbilliviral epizootic in Atlantic bottlenose dolphins of the Gulf of Mexico. J Vet Diagn Invest. 1996 (in press).
14. Visser IKG, van Bressem M-F, de Swart RL, van de Bildt MWG, Vos HW, van der Heijden, RWJ, et al. Characterization of morbilliviruses isolated from dolphins and porpoises in Europe. J Gen Virol 1993;74:631-41.
15. Kenney RD. Bottlenose dolphins off the northeastern United States. In: Leatherwood S, Reeves RR, editors. The bottlenose dolphin. San Diego: Academic Press, 1990:369-86.
16. Breslow NE, Day NE. Statistical methods in cancer research.Vol. 1 Lyon, France. International Agency for Research on Cancer, 1980.
17. Geraci JR. Clinical investigation of the 1987-88 mass mortality of bottlenose dolphins along the US central and south Atlantic coast: final report to National Marine Fisheries Service and U.S. Navy. Guelph, Canada: Office of Naval Research, and Marine Mammal Commission; 1989.
18. Duignan PJ, House C, Geraci JR, Duffy N, Rima BK, Walsh MT, et al. Morbillivirus infections in cetaceans of the western Atlantic. Vet Microbiol 1995;44:241-9.
19. Duignan PJ, House C, Geraci JR, Early G, Copland H, Walsh MT, et al. Morbillivirus infection in two species of pilot whales (*Globicephala* sp.) from the western Atlantic. Mar Mam Sci 1995;11:150-62.
20. Reid AH, Tsai MM, Venzon DJ, Wright CF, Lack EE, O'Leary TJ. *MDM2* amplification, *P53* mutation, and accumula-tion of the *P53* gene product in malignant fibrous histiocytoma. Diagn Mol Pathol 1996;5:65-73.
21. Kovalic D, Kwak J-H, Weisblum B. General method for direct cloning of DNA fragments generated by the polymerase chain reaction. Nucleic Acids Res 1991;19:4560.
